# Safety and practicality of an excisional lymph node study driving HIV cure research in South Africa

**DOI:** 10.3389/fimmu.2024.1442556

**Published:** 2024-08-26

**Authors:** Trevor Khaba, Andrea Olga Papadopoulos, Thandeka Nkosi, Sifundo Nxele, Thandekile Ngubane, Ismail Jajbhay, Johan Pansegrouw, Zaza M. Ndhlovu

**Affiliations:** ^1^ Human Immunodeficiency Virus (HIV) Pathogenesis Programme, The Doris Duke Medical Research Institute, University of KwaZulu-Natal, Durban, South Africa; ^2^ Africa Health Research Institute, Nelson R. Mandela School of Medicine, University of Kwa-Zulu Natal, Durban, South Africa; ^3^ KwaZulu-Natal Department of Health, Prince Mshiyeni Memorial Hospital, Durban, South Africa; ^4^ Ragon Institute of Massachusetts General Hospital, Massachusetts Institute of Technology, and Harvard University, Cambridge, MA, United States

**Keywords:** lymph node excision, HIV cure, tissue immunology, HIV reservoir, lymph node metastasis

## Abstract

**Introduction:**

Studying diseased human tissues offers better insights into the intricate interactions between pathogens and the human host. In conditions such as HIV and cancers, where diseases primarily manifest in tissues, peripheral blood studies are limited in providing a thorough understanding of disease processes and localized immune responses.

**Methods:**

We describe a study designed to obtain excisional lymph nodes from volunteers for HIV reservoir studies. Since study commencement in 2015, 181 lymph node excisions have been performed, resulting in collection of 138 lymph node tissues. Lymph nodes were surgically excised from study volunteers using a minimally invasive procedure, performed in a minor theater under local anesthesia.

**Results:**

The surgery takes less than 30 minutes to complete, minimizing risk and stress on the volunteer. The small incision made during the procedure typically heals within a week. The associated discomfort is generally manageable, and participants are often able to resume their regular activities within a day. Only 5.5% of the study participants experienced minor adverse events, such as swelling and prolonged wound healing, recovering within 2 weeks with no serious adverse events reported.

**Discussion:**

Our study demonstrates that when done with outmost care, obtaining excised lymph nodes for research is relatively safe and practical.

## 1 Introduction

Basic human immunology research has contributed to our understanding of human diseases and led to the development of many life-saving medical therapies and vaccines. However, the major limitation of human immunology is the over-reliance on peripheral blood samples for research due to the ease of peripheral blood sampling. Accessing lymph nodes for research would greatly enhance the field of immunology as these organs exist as hubs for crucial adaptive immune responses, such as antigen presentation for antibody production within lymph node germinal centers, as well as key sites of autoimmune regulation. Although human tissue samples are not easily accessible for research, tissue studies have provided invaluable insights into the molecular and cellular mechanisms underlying diseases. Studying infected tissues can help to identify specific interactions between pathogens and the host cells, uncover disease progression patterns and elucidate factors influencing infection severity and disease outcomes. The discovery of tissue resident immune cells that are phenotypically unique and distinct from circulating immune cells has led to increased interest in studying immune responses in native tissue sites (1, 2). For instance, direct solid tumor tissue analysis has revealed the important role of tissue resident CD8^+^ T cells within tumors in diseases outcomes (3–5). Knowledge from tissue studies is crucial for the development of effective diagnostic tools, treatments, and preventive strategies.

In our research aimed at achieving a cure for HIV, human tissues have proven essential in elucidating the location of HIV reservoirs and understanding their persistence within lymphoid tissues (6–9). Animal models have offered valuable insights into HIV reservoir dynamics due to their potential for controlled experimentation, extensive tissue sampling, and longitudinal studies (10). However, since HIV primarily infects humans, animal models like Non-Human Primate models (NHPs) can simulate some aspects of HIV infection but cannot fully replicate the complexity and dynamics of HIV reservoir formation and persistence in humans. Species-specific differences limit the direct translation of findings from animal models to humans (11–13).

Although obtaining human tissue samples requires invasive procedures, the benefit of obtaining excised lymph nodes for HIV reservoir research outweigh the risk associated with the minor surgical procedure involved. Excised lymph nodes samples are particularly advantageous because they yield large quantities of material that can be processed in a variety of ways. This offers greater opportunities for researchers to conduct more detailed characterization of the HIV reservoirs at a tissue level.

Excisional lymph node procedures are challenging to perform due to ethical considerations and the need for extensive consent and the important need to prioritize the safety and wellbeing of study participants. A less invasive alternative is fine needle aspiration (FNA), which allows direct sampling of lymph node tissue cells (14). This technique enables direct assessment of immune cell populations, activation status, and the molecular signatures within lymph nodes. Although FNAs have proved useful for evaluating immune responses to vaccines, they have several limitations. Firstly, FNA samples represent a small portion of the lymph node tissue and do not fully capture the heterogeneity of immune responses within the entire lymph node. Secondly, FNA samples have limited cellularity which severely limits downstream assays that can be performed, and the sample size is often insufficient for cryopreservation. Thirdly, FNA samples are prone to carry-over contamination or infection during sample collection (15, 16). Fourthly, FNA samples do not capture spatial information which is vital for understanding HIV tissue reservoirs.

Given the importance of lymph node tissue studies in HIV reservoir studies for cure, our group has overcome the barrier of access to clinically relevant lymph node samples by establishing a Lymph Node study at our study site in Durban, South Africa. We leveraged collaborative networks with physicians/surgeons, community leaders and People Living with HIV (PLWH) to establish the infrastructure and capacity to collect excised lymph node tissue samples from PLWH and healthy controls. Our tissue sample repository that we have accumulated over the years has uniquely positioned us to carry out studies that provide a deeper understanding of HIV-1 reservoirs and immune responses within native tissue environments (6, 9). We also share the samples with collaborators across the world.

## 2 Study design

### 2.1 Study sites and ethics approval

The lymph node study housed at the University of KwaZulu Natal was established in 2015 after extensive stakeholder discussions and rigorous Ethics review process by the Biomedical Research and Ethics Committee (BREC), UKZN and Massachusetts General and Brigham (MGH) Hospital review boards, including input from Community Advisory Boards. Participants were recruited as described below. Surgeries were performed at Prince Mshiyeni Memorial Hospital, KwaZulu Natal, South Africa.

### 2.2 Recruitment procedure and informed consent

At the time of enrolment all new participants are assigned a unique research identification number. Eligible participants who are currently enrolled in other studies are enrolled using a new Lymph node study research identification number. All participant information is linked and tracked through these respective research identification numbers. Participation into this study is voluntary and participants may choose to withdraw at any time. Our lymph node study comprises two recruitment arms. Arm 1 is opportunistic and seeks to enroll participants from individuals already seeking care at Prince Mshiyeni Memorial Hospital, a tertiary health facility in KwaZulu Natal. We target individuals scheduled for lymph node excision for either curative or diagnostic purposes. On the morning of the surgical procedure, the study nurse reaches out to potential volunteers to provide study details. If they express interest and meet the eligibility criteria, informed consent is obtained, and a blood sample is collected before surgery. During the procedure, the surgeon extracts an additional lymph node specifically for our study as described under ‘lymph node excision’. Participants in this arm are only seen on the day of the procedure, any further follow-up visits are arranged through regular clinical care. Clinical demographic data pertinent to our study is gathered from the participants and their primary physician. Arm 2 consists of participants recruited from other ongoing prospective cohorts of PLWH, particularly the “Females Rising with Education, Support, and Health (FRESH) (17), “Characterization of the evolution of adaptive and innate immune responses in acute HIV clade C virus infection” cohort and the “Immunology and Virology of HIV Controllers” cohort. These are all studies that fall under the HIV Pathogenesis Programme (HPP). The FRESH and HPP Acute studies are interested in the pathogenesis of clade C HIV infection, specifically aimed at identifying individuals who are acutely HIV positive (within a few months of HIV seroconversion). The Elite Controller study involves the enrolment of HIV elite controllers, a subset of PLWH who achieve spontaneous control of viral replication for prolonged periods in the absence of treatment. Acute and Elite cohorts. These participants are recruited to undergo a lymph node excision procedure solely for research purposes. During the first visit the participants are screened for eligibility and once enrolled are booked for lymph node excision and blood draw. The participants are then followed up for 1 additional visit where a review of their clinical condition and recovery will occur.

### 2.3 Participant eligibility criteria

#### 2.3.1 Arm 1

The eligibility criteria include 1) both male and females above 18 years, 2) willing and able to give written informed consent, and meet the study eligibility criteria, 3) Any type of lymph node scheduled for excision will be accepted. The exclusion criteria include 1) history of underlying medical conditions for which antibiotic prophylaxis for invasive procedures was required, 2) received an experimental HIV vaccine, has overt cancer, active tuberculosis disease or any infectious diseases that would render the sample unsafe for processing in bio-safety level 2 lab. These are determined through the patient’s hospital records. 3) female subject who are pregnant determined through a urine pregnancy test (sensitive to 25 IU HCG) at screening and within 24 hours of the study procedure or less than 8 weeks post-partum.

#### 2.3.2 Arm 2

Study eligibility criteria include: 1) males or females, between 18 to 49 years of age, 2) Ability and willingness to give written informed consent and to comply with study requirements, 3) Individuals living with HIV who have no evidence of opportunistic infection or malignancy or HIV negative individuals with documented HIV negative results, 4) Any type of lymph node scheduled for excision will be accepted, 5) a negative result for a urine pregnancy test (sensitive to 25 IU HCG) at screening and within 24 hours of the study procedure for female participants, 6) Point of care or lab values within 28 days prior to enrolment of Hemoglobin > 10.0 g/dL, 7) Safety blood tests performed will be a Full Blood Count, Urea and Electrolytes and International Normalized Ratio. Exclusion criteria include 1) Women who are pregnant or less than 8 weeks post-partum, 2) recent history of use of any immunomodulatory agents within 30 days prior to study enrollment; 3) History of sensitivity, allergy or anaphylaxis to benzodiazepines or other narcotics that are used during the procedure, 4) Previous adverse reaction or allergy to lidocaine or other amide anesthetics, as well as benzocaine or other ester type anesthetics, 5) History of underlying medical conditions for which antibiotic prophylaxis for invasive procedures is required, 6) History of coronary artery disease, myocardial infarction, COPD, chronic renal failure, decompensated cirrhosis, or any other condition that in the opinion of the investigator will compromise ability to participate in the study, 7) Currently taking anticoagulants including but not limited to: heparin (Hep-Lock, Hep-Pak), Hep-Pak CVC, Heparin Lock Flush), warfarin (Coumadin), tinzaparin (Innohep), enoxaparin (Lovenox), danaparoid (Orgaran), dalteparin (Fragmin), clopidogrel (Plavix), dipyridamole (Persantine), fondaparinux (Arixtra), argatroban (Agratroban), bivalrudin (Angiomax), prophylactic aspirin, and regular NSAID use, 8) Participants taking any of the following medications: systemic steroids (inhaled or nasal steroid therapy permitted), interleukins, systemic interferons (e.g. local injection of interferon alpha for treatment of HPV is permitted) or systemic chemotherapy, 9) Prior recipient of HIV vaccine.

Individuals who meet the eligibility criteria are informed about the risks and benefits of the study. If they choose to participate voluntarily, the informed consent procedure is administered.

Phlebotomy: Phlebotomy is done by a trained phlebotomist using standard venipuncture technique. All study research specimens are labelled with the participants unique research identification number. Prior to each phlebotomy performed for the study, a participants is asked questions regarding their health status, recent fever, weight loss, and possible pregnancy. Most recent hemoglobin test results are verified prior to phlebotomy. If the previous hemoglobin was less than 10.0 g/dl the total quantity of blood drawn is limited to 50 mL for study-related clinical and research labs. The amount of blood collected from these individuals will not exceed 50 mL or 3 mL per kg, whichever is less, in an 8-week period taking into consideration the age, weight and health of the participants. Participants may refuse phlebotomy at any time. Not more than 120mL of blood is collected for each healthy participant.

### 2.4 Pre-excision safety protocol

After recruiting participants, the study physician conducts safety investigations prior to the surgery. These investigations include a comprehensive physical examination and obtaining a blood sample for laboratory tests, including Full Blood Count (FBC), international normalized ratio blood test (INR), Urea & Electrolytes (U&E), Hemoglobin (must be greater than 10.0 g/dL), CD4 count, and HIV Viral Load. The surgeon schedules the participant for the procedure only if the physical exams and lab tests meet the safety criteria. If any of the results fall below acceptable ranges or if the participant is clinically unwell, the surgery is either cancelled or postponed. Additionally, participants are required to complete a disclosure form before surgery, providing details about their current health status, body weight, and recent blood draws.

### 2.5 Excisional lymph node surgical procedure

The Lymph node excision is a relatively safe procedure performed in the minor theatre under local anesthetic. A qualified, board-certified surgeon performs the procedure after ensuring that the physical assessments and lab tests meet the safety threshold outlined above. In preparation for surgery the participants are advised not to eat or drink for 8 hours before the procedure. Once in theatre, intravenous access is established by the surgical team. The surgeon uses their discretion to refer participants who have small or deeply set lymph nodes that are difficult to palpate to the radiology department. The radiology department assists in identifying the lymph node location under ultrasound guidance and mark the location on the skin. This is done to limit the incisions and decrease the need for lymph node exploration. The surgeon palpates for the node and marks the area where a node is identifiable. The participant is then premedicated with an IV anxiolytic and/or opioid prior to the procedure according to standard medical practice. The excision region is prepared with a sterilizing solution and draped with sterile towels. A single-dose of IV antibiotics is administered. The area is then anesthetized with a 1:1 combination of 1% lignocaine and 0.5% Marcaine to provide immediate and long-lasting local anesthesia 4 cm incision is made overlaying the nodal basin using a diathermy pencil and the node is excised out. The wound is sutured with nylon 3.0, approximately only 3 stitches are made. Ligatures are placed on lymph vascular pedicles where necessary. The wound is closed in layers using absorbable suture material and a standard dry sterile dressing is applied before the patient is taken out of theatre to the post-operation observation room. To prevent participants from experiencing excessive pain and discomfort, lymph nodes situated near nerves are not excised.

### 2.6 Post-procedure patient safety and adverse events

Participants from arm 2 are monitored routinely by the HPP study staff in Umlazi. They are educated on wound care and provided with pain medication. Approximately two weeks following the procedure (± 2 days), they are booked for a follow up with a study doctor or nurse. During this visit the surgical site is assessed and the participant is asked about post procedure recovery. If any concerns are raised the participant is referred to the surgical department for follow up. If the complication is severe and requires additional surgery, the study will cover the cost. If the participant develops complications before their scheduled follow up visit, then they are encouraged to contact the study nurse to arrange a follow up.

Participants from Arm 1 are given instructions on post operative care or the wound and on pain management from a member of the surgical team. Those that received only local anesthetic are discharged from the recovery room to home once they are awake and stable. Participants who received general anesthesia continue to be monitored for a period of at least 6 hours post procedure in the recovery ward before being discharged. Over the counter pain medication is provided and the participants are advised to contact the study nurse if pain is not adequately relieved or if there are any signs or symptoms of infection, bleeding, or worsening discomfort. If an adverse event does occur the study nurse takes the participants back to the surgical department where they are evaluated, diagnosed and treated for any surgical site infection. The signs that the study nurse looks out for include but are not limited to; dark colored stool, shortness of breath, severe and persistent abdominal pain, chest pain or vomiting blood. If they exhibit any of these symptoms they will be asked to return to the Hospital immediately.

Our study prioritizes the well-being of participants, ensuring that any adverse events are promptly addressed and managed by our experienced medical team. Our adverse event monitoring and resolution plan include the following contingencies: 1) We convene monthly meetings focused on the operations of the study and constantly remind our medical team to remain vigilant in monitoring participants for any signs of adverse events during and after surgeries; 2) Participants are provided with comprehensive education regarding potential risks and the importance of promptly reporting any discomfort or unusual symptoms following surgery; 3) We have a comprehensive post-operative follow-up procedure which involve daily phone calls with the participants to optimize timely assessment and management of any adverse events that may arise; 4) Once an adverse event occurs, our research team conducts a thorough review of study protocols to identify any areas for improvement to minimize similar future adverse events. Participants in Arm 1 are at a slightly higher risk than Arm 2 participants because they are booked for the procedure for other purposes. There may be a larger incision and exploration at the surgical site due to excision of the additional lymph nodes for research purposes. This may cause additional trauma and delay recovery. Due to more lymph node tissue being removed they are also at a higher risk in the long term for lymphoedema and lymphocele, which may result from extensive lymph node removal.

### 2.7 Lymph node sample processing and storage

Following the excision procedure the lymph node is placed in R10 media [RPMI 1640 medium supplemented with heat-inactivated fetal bovine serum (FBS), 1% penicillin/Streptomycin, HEPES and 1% L-glutamine (Gibco, Schwerte, Germany)] and kept on ice between 0C° and 4C° and transported to the lab within an hour post-excision. Once the lymph node arrives in the lab, it is immediately placed in a petri dish with R10 media and processed according to the method developed by Schacker et al. (18) and similar to Schleimann et al. (19). Briefly, sterile forceps and a disposable scalpel are used to trim off the fat surrounding the node. After cleaning the node, a piece of about a third of the node is cut off and immediately placed in fixing buffer (10% Neutral buffered formalin) for tissue wax embedding and downstream immunohistochemistry work. Additional media is added after which the node is mechanically disintegrated using scalpel and forceps. The macerated tissue is suspended in media and passed through a cell strainer (70 µm) to collect lymph node mononuclear cells and granulocytes (collectively LMC) into a conical tube. The LMC are washed twice in R10 media and either used immediately in an experiment or cryopreserved in freezing media (20% DMSO in FBS) in liquid nitrogen at around -195C°.

## 3 Results

**Figure 1:**
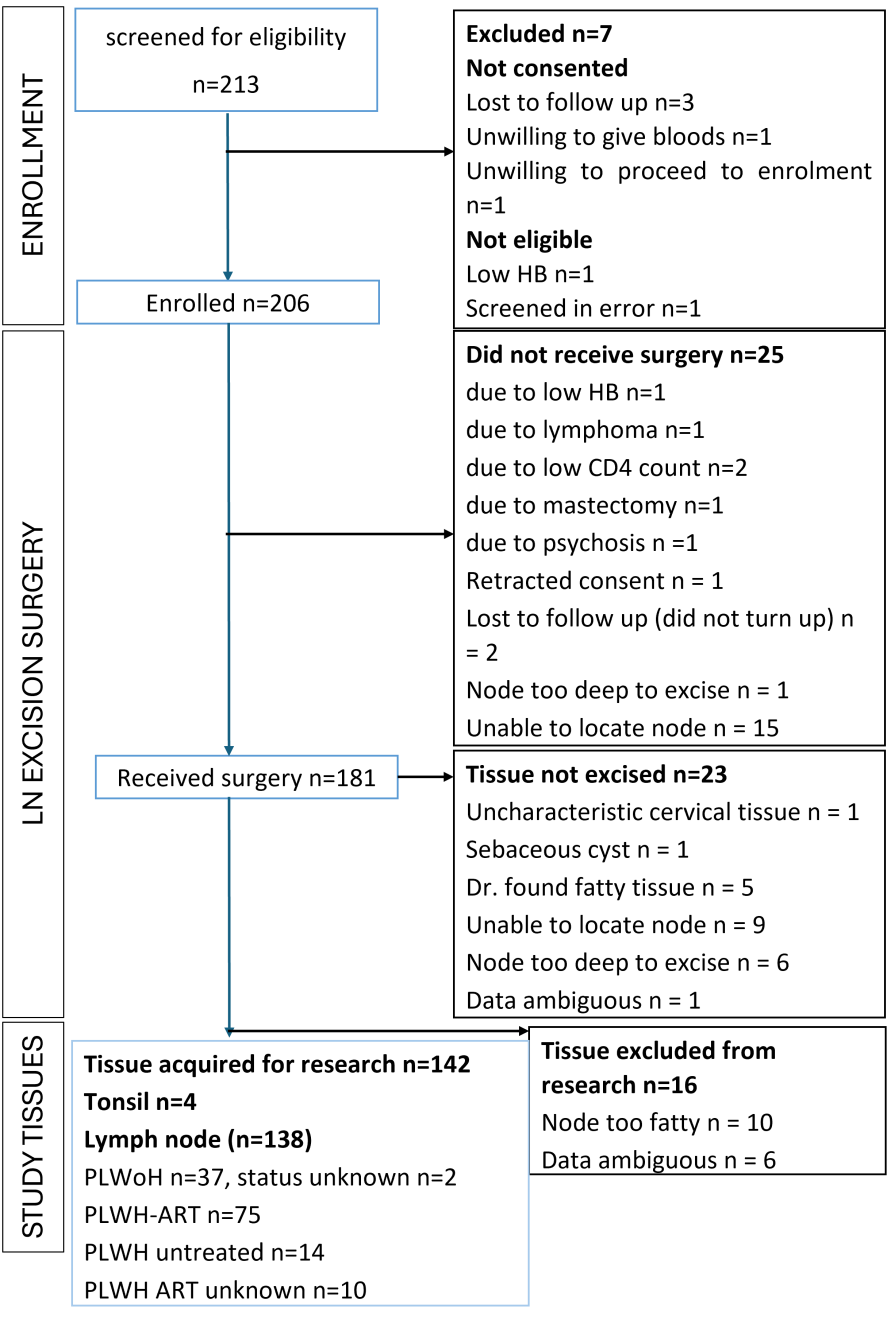
Participant Screening and Enrollment Flowchart. This flowchart outlines the screening and enrollment process of participants in the study. A total of n=213 individuals were screened of which n=206 participants were enrolled into the study. Of the enrolled participants, 181 underwent lymph node surgery as part of the study. Following successful surgery, n=142 of these samples were identified as usable lymph node tissues, excluding those consisting solely of fat tissue.

A total of 206 participants have been enrolled into the study between 2015 and 2022 (**Figure 1**; **Table 1**). After enrolling 181 surgeries, 138 lymph node tissues (76%) obtained were usable for downstream research (**Figure 1**). Demographics and clinical parameters of the 138 participants are summarized in **Table 1**. 25 enrolled participants did not meet pre-surgery screening criteria, retracted consent or were lost to follow up prior to surgery (**Figure 1**). Common challenges that the surgical team has encountered during this study included: 1) Lymph nodes that were too deep to be excised safely such that, local anesthetic might not be sufficient. These lymph nodes are not excised. 2) In certain cases, surgeries were cancelled due to proximity of LN to nerves or other health considerations of the participant (low Hb or low CD4 count) according to the safety protocol (section 2.4). 3) Occasionally, fat and other tissue obscures the lymph node, yielding only fatty tissue biopsies, which is only identifiable post- procedure. This occurred in 10% of the biopsies performed.

**Table 1:** Demographics and clinical parameters of participants from whom LN were successfully obtained (n = 138).

	n	Arm	Age (Med)	Gender	CD4 count (Med)	VL (unsupp. range x10^3^)	node		
							Inguinal	Axillary	Cervical
**All**	138	1:10 2:128	18-69 (22)	F:130 M:8	105-1801 (726)	Supp: 47; unsupp 44 (0.044-400)	122	7	9
**PLWoH**	37	1:3 2:34	18-69 (22)	all F	659-1333 (938)	nd	30	2	5
**PLWH on ART**	75	1:2 2:73	18-40 (22)	F: 72 M: 3	105-1801 (701)	Supp: 43; unsupp 30 (0.044-170)	71	3	1
**PLWH untreat.**	14	1:2 2:12	18-51 (24)	F:12 M:2	251-1273 (638)	Supp: 1; unsupp: 13 (0.24-400)	10	1	3
**PLWH ART nd**	10	1:1 2:9	21-58 (27)	F: 9 M:1	430-1683 (850)	Supp: 3; unsupp 1 (7.3)	10	–	–
**HIV nd**	2	1	56, 65	F1, M1	nd	nd	1	1	-

Med: median; CD4 count: cells/µl (not determined for n=24); VL: viral load copies/ml (not determined for n=8). Unsupp: >40 copies/ml; supp: undetectable viral load (<40 copies/ml).

Post-lymph node excision, the adverse events that have occurred between 2015 and 2022 encompassed 5.5% of surgeries (10/181) and are further described in **Table 2**.

**Table 2:** Adverse events reported for lymph node excision surgery during the Lymph Node Study from 2015- 2022.

Adverse Event Detail	Type of node	Year reported	Action taken	Duration to recovery	Age	HIV status	Gender
Returned with chest pains	Cervical	2016	Participant was diagnosed with TB, was started on TB treatment, successfully completed treatment – fully recovered.	6 months TB treatment	24	Positive	Female
Swollen wound	Inguinal	2016	Antibiotics and painkillers were administered to participant. At one week follow-up, wound healed and no further complaints	21 days	22	Positive	Female
Swollen wound	Inguinal	2017	Wound redressed, antibiotics and painkillers administered. wound healed at 1-week follow-up visit	18 days	21	Positive	Female
Fever pain and swollen wound site	Inguinal	2018	Antibiotics were given and wound redressed. Counselling revealed defaulting on ART treatment. Participant was re-educated on ART adherence and seen weekly for one month till wound healed.	1 month	21	Positive	Male
Swollen wound	Inguinal	2018	Participant given antibiotics and painkillers, seen weekly until wound healed for 2 weeks.	23 days	19	Positive	Female
Swollen wound	Inguinal	2018	Wound redressed with no signs of infection. At one week follow-up, wound had healed.	22 days	21	Positive	Male
Pain and serous fluid on the excision wound	Inguinal	2018	Antibiotics and painkillers given to participant. Wound redressed. At follow-up, wound had healed and sutures were removed.	22 days	21	Negative	Female
Swollen wound accompanied by erectile dysfunction	Inguinal	2018	Participant had healed well but reported occasional pain from wound site and erectile dysfunction. Counselling was done followed by referral to reproductive men’s clinic. At 3 month follow-up, participant had recovered.	5 months	22	Positive	Male
Pain and swollen wound	Inguinal	2019	Antibiotics and pain killers were given. Wound redressed. At follow-up, wound had healed.	23 days	21	Negative	Female
Pain and swollen wound	Inguinal	2019	Antibiotics and pain killers were given. Wound redressed. At follow-up, wound had healed.	23 days	23	Positive	Female

**Figure 2:**
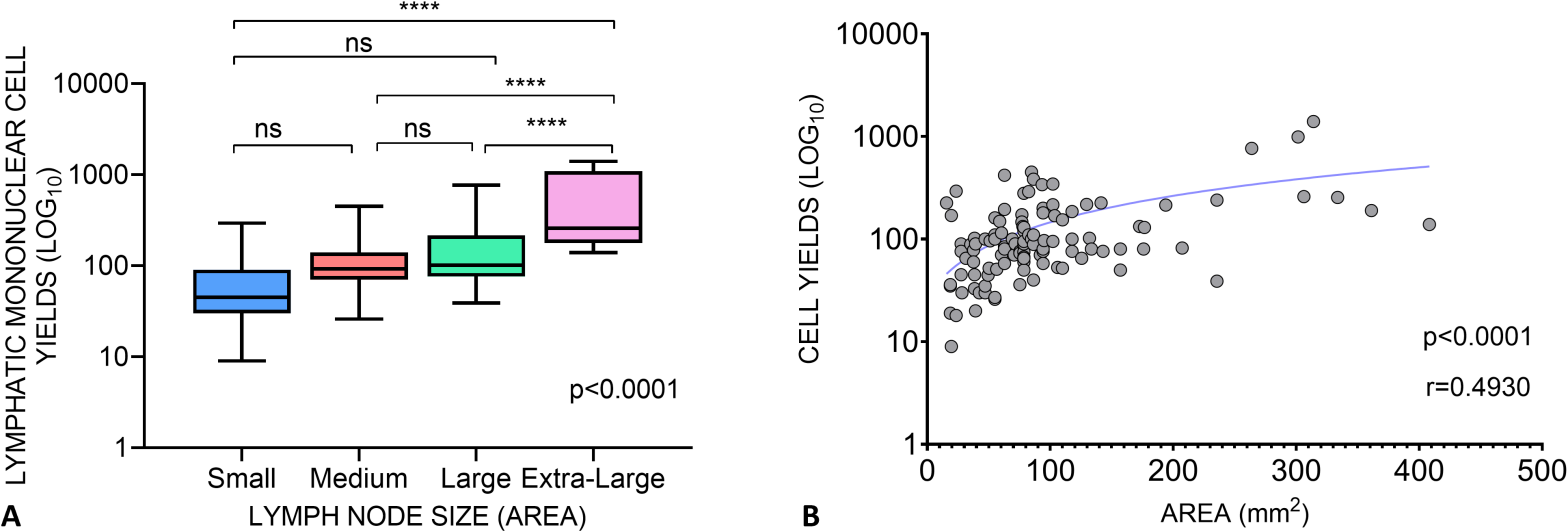
Lymphatic Mononuclear Cell Yields from lymph nodes acquired in The Lymph Node Study. **(A)** Cell yields of Lymph node tissues grouped as Small (<50 mm2), Medium (>50-100mm2), Large (>100-300mm2) and Extra Large (>300mm2) based on the product of their length and width. One-way ANOVA shows a significant difference in cell yields between the different lymph node size groups (ANOVA, p < 0.0001). The Tukey’s multiple comparisons test was performed between the groups: Small vs. Medium p=0,4880, Small vs. Large p=0,2121, Small vs. Extra Large p<0,0001, Medium vs. Large p=0,8350, Medium vs. Extra Large p<0,0001, Large vs. Extra Large p<0,0001. **(B)** Relationship between the area of lymph nodes and the yield of lymphatic mononuclear cells. The Pearsons correlation coefficient r=0.4930 and p<0,0001 suggests a significant positive association between the size of the nodes and the LNMC yield. **** means p<0.0001. ns, not significant.

Immediate and quick processing of the lymph node upon arrival in the lab is integral to attaining good cell yields. Following the method established by Schacker et al. (18), ensures a tested and standardized approach which is essential for maintaining the integrity of the samples and obtaining reliable results in immunological and pathological investigations downstream. To achieve this, the lymph node is delicately handled, with sterile forceps and a disposable scalpel used to remove the surrounding fat. This step is crucial for obtaining a clear view of the node tissue and ensuring a good yield of cells. Following the initial cleaning, a portion of the node, typically a third is excised and promptly placed in neutral buffered formalin. This preservation technique facilitates tissue wax embedding, preserving the structural integrity of the node for subsequent immunohistochemistry investigations. The node is mechanically disintegrated and then filtered through a cell strainer to isolate lymph node mononuclear cells and granulocytes (collectively LMC), which are either used immediately in experiments or cryopreserved in freezing media before storage in liquid nitrogen. The greatest advantage of excisional lymph node biopsy is the large number of cells that can be obtained, enabling more in-depth scientific investigations. In our study, we received lymph nodes of varying sizes, ranging from 5 mm to 30 mm. When comparing the size of the whole LN, and the yield of cells obtained after macerating 2/3 of the node, the smaller nodes, which were ranged from 15.7mm^2^ to 49.5mm^2^ yielded a median of 45 million cells (IQR of 60 million); medium nodes ranged from 50.24mm^2^ to 94.99mm^2^ and yielded a median of 92 million cells (IQR of 70 million); the larger nodes ranged from 102.05mm^2^ to 263.76mm^2^ and yielded a median of 101 million cells (IQR of 139.3 million); the extra-large nodes ranged from 301.44mm^2^ to 408.2mm^2^ and yielded more, with a median of 257.5 million cells (IQR of 915.7 million) (**Figure 2A**). The area of the node correlated with mononuclear cell yield (**Figure 2B**).

## 4 Discussion

We have established logistical infrastructure and functional collaborations with surgical teams, community advocates, and our clinical and basic scientist teams to streamline the recruitment of volunteers willing to donate excisional lymph nodes for research. The success of our program is attributed to rigorous pre-surgery screening and utilizing a minimally invasive procedure that only requires local anesthetic. Participant safety is further bolstered through post-surgery monitoring aimed at early detection of potential adverse events.

One of the primary benefits of acquiring excised lymph nodes is the opportunity to study human diseases within their natural tissue environments. While animal models have significantly advanced our basic understanding of disease processes, leading to the development of new therapies, intrinsic differences between animal and human hosts affect model translation. Mice for example have limited ability to produce certain important inflammatory cytokines (11) and exhibit significant differential transcription responses to disease and injury relative to humans (12). Moreover, animal models have limitations when it comes to infectious pathogens like HIV, which specifically infect humans (20).

In our research, a single excised node can be divided into three or more pieces that can undergo different processing methodologies. For example, one piece can be snap frozen for downstream imaging approaches, another piece is fixed and later used in variety assays and one more piece can be used to isolate a high number of cells for downstream assays that utilize single cell suspensions. The opportunity to perform a variety of investigations on the same sample increases the potential to uncover detailed mechanisms responsible for viral persistence and guide the development of precise strategies for targeting the reservoir for elimination.

A notable advantage of tissue studies is the opportunity for conducting spatial biology investigations. This is crucial for understanding how the spatial characteristics of tissues influence immune responses and the clearance of pathogens. For instance, we and others have utilized lymph nodes to shed light on why lymph node B cell follicles serve as sanctuary sites for HIV even during antiretroviral therapy (ART)-mediated suppression (3, 6, 7, 9, 10, 21). Image analysis following immunohistochemistry and *in situ* hybridization of lymph nodes obtained from individuals suppressed by ART reveals that the exclusion of CD8^+^ cells from the germinal centers, primarily due to limited expression of CXCR5, is a major contributing factor to why the immune system fails to clear the follicular HIV reservoir (9). In a recent TLR9-agonist trial (MGN1703), safe, LN biopsies served to localize CD8 T cell activation within the LN of treated participants, with promising implications for enhancing anti-HIV cytolytic responses within these tissues (19). The advent of spatial transcriptomics and proteomics has also provided a new perspectives of tissue immunology, with numerous studies of tumor lymph node tissues shown to be feasible (22–26), provided tissues are fixed within hours of biopsy, and transcriptomics assays performed within 3 years of embedding before RNA begins degrading (27). Recently, excisional lymph nodes have been used to show how poor germinal center formation results in severe Covid-19 diseases (28).

Human tissue studies, while immensely advantageous for advancing human health research, pose challenges in their execution and management, including safety concerns and challenges in recruiting study volunteers due to perceived risks associated with surgical procedures. However, our study illustrates that when conducted appropriately, lymph node excision for research purposes is relatively safe, feasible, and provides valuable samples for spatial biology studies. It is noteworthy that our experience is similar to other groups that have also safely conducted lymph node excision procedures for research purposes. For example, a study conducted in rural Tanzania involving inguinal lymph node biopsies reported no complications in 7 out of 9 participants, with a recovery period of up to 2 weeks, with two participants experiencing self-resolving, hyperemia (29). Additionally, a Ugandan HIV study obtained 138 inguinal lymph node (LN) biopsies from 71 participants some of whom underwent three repeat biopsies, with only a handful episodes of lymphoceles (30). Furthermore, a Thailand cohort reported mild events of self-resolved site discomfort in 12.8% of participants (31). Of all the studies reviewed in the literature, only one study done in the US reported post-surgery infection (32).

Risk of lymphedema following LN excision is well documented in the cancer field. The risk of complications following inguinal lymph node excision is approximately 4%, and following axillary lymph node excision is approximately 7% (33, 34). The risk of bleeding at the site is less than 3%, and the risk of formation of a post-operative fluid collection (seroma) is less than 7%. Fluid at the post-operative site most often can be managed without surgical intervention, but the surgeon may decide to pursue open or percutaneous drainage based upon the size of the collection and any associated symptoms. The risk of infection at the biopsy site is less than 4%. If a surgical wound infection develops the patient is prescribed antibiotics. Additionally, at the surgeon’s discretion, the wound may need to be re-opened and treated with twice-daily dressing changes for one to two weeks. The patient may experience minor swelling and pain at the site of the incision. The pain usually persists for 1-5 days but in rare circumstances can persist much longer. Paracetamol or ibuprofen is recommended to help with the pain. There is a small risk (<1%) that the patient will have chronic nerve dysfunction, pain, or peri-incisional loss of sensation (which may extend over the lateral upper arm in the case of axillary lymph node biopsy). In addition, there is <2.5% risk that chronic swelling may persist in the post-operative period. In the event of extensive removal or destruction of lymphatic tissue, there is a long-term risk of lymphoedema and/or a lymphocele. There is a risk of anaphylaxis and reaction to the drugs used intraoperatively and perioperatively (local anesthetics, antibiotics and general anesthesia).

Considering the highlighted benefits of studying whole human lymph node tissues for understanding diseases and developing cures and vaccines, we propose the following recommendations for researchers aiming to establish their own lymph node studies. Firstly, it is essential to recognize that lymph node excisions for research purposes constitutes high-risk human subject research, and therefore necessitates a rigorous and extended ethics review process. This is crucial to ensure that all necessary safety measures are in place to minimize harm to the participants. Consequently, meticulous planning prior to the commencement of the study is of paramount importance. The initial step involves securing funding for the study. Secondly, it is imperative to identify a surgeon willing to serve as a substantive collaborator on the project, preferably, a senior surgeon capable of mobilizing a surgical team to be part of the study. Thirdly, obtaining the endorsement of community representatives from the target study population. This entails sharing detailed information with the community about the significance of the study, explaining the associated risks, and outlining the risk mitigation plan. In our case, it was essential to ensure that members of the Community Advisory Board (CAB) representing people living with HIV not only endorsed the project but also provided input into the study design (35). Fourthly, it is essential to establish a clinical study team that works closely with research laboratory teams and ethics experts. This collaborative approach ensures efficient coordination and adherence to ethical standards throughout the study. When drafting study protocols for submission to the ethics review board, it’s imperative that the entire team, including surgeons, bioethicists, lab team members, and community representatives, collaboratively contribute to putting together the document. The ethics application must provide a clear outline detailing how participant safety will be ensured at each stage of the study.

Lastly, ensuring the acquisition of high-quality tissue samples demands meticulous attention to every stage, starting from participant recruitment to sample processing and storage. In our study, we optimized the entire process, including sample collection, transportation, processing, and storage using excess lymph nodes left over from diagnostic histology procedures. Additionally, identifying lymph nodes in healthy individuals can pose challenges, especially for inexperienced surgeons, as the nodes are typically small and situated deep within tissues. Based on our experience, ultrasound technology was invaluable for precisely locating nodes and avoiding fat tissue collection. In all cases, ultrasound-guided lymph node excision were successful.

In our associated FRESH cohort, a study was conducted to determine participant and community perceptions of the study (35). Here, perceptions were generally positive, demonstrating that our study environment is a safe place for open discussion about risks and benefits of participating in research studies (35). For our study, we acknowledge that the safety data is limited to events reported by participants. It is possible that some participants may not feel comfortable raising concerns; however, this is speculative and requires more formal social studies to substantiate. It is also important to note most of our participants were female, who are more likely than males to seek medical care and likely more willing to volunteer for such studies (35). It is noteworthy that 3 of the 8 male participants (37%) reported mild adverse events, compared to 4% of females. This may suggest that males are more likely to report adverse events than females. However, studying the impact of gender on adverse event reporting is challenging in this context due to the small number of male participants compared to females. Occurrence of adverse events based on gender will be addressed in future studies, where we plan to recruit more males.

In summary, we successfully established a study to obtain lymph node tissues from the region in South Africa most-heavily impacted by the HIV epidemic. We found the process, including safe and simple surgery, to be highly valuable for spatial characterization of HIV tissue reservoirs which is currently the greatest barrier to HIV cure, thus advancing the field of HIV and spatial immunology.

## Data Availability

The raw data supporting the conclusions of this article will be made available by the authors, without undue reservation.
